# Feeding Mechanics in Spinosaurid Theropods and Extant Crocodilians

**DOI:** 10.1371/journal.pone.0065295

**Published:** 2013-05-28

**Authors:** Andrew R. Cuff, Emily J. Rayfield

**Affiliations:** School of Earth Sciences, University of Bristol, Bristol, United Kingdom; Raymond M. Alf Museum of Paleontology, United States of America

## Abstract

A number of extant and extinct archosaurs evolved an elongate, narrow rostrum. This longirostrine condition has been associated with a diet comprising a higher proportion of fish and smaller prey items compared to taxa with broader, more robust snouts. The evolution of longirostrine morphology and a bulbous anterior rosette of premaxillary teeth also occurs in the spinosaurid theropod dinosaurs, leading to suggestions that at least some members of this clade also had a diet comprising a notable proportion of fish or other small vertebrates. Here we compare the rostral biomechanics of the spinosaurs *Baryonyx walkeri* and *Spinosaurus* c.f. *S*. *aegyptiacus* to three extant crocodilians: two longistrine taxa, the African slender-snouted crocodile *Mecistops cataphractus* and the Indian gharial *Gavialis gangeticus*; and the American alligator *Alligator mississippiensis*.

Using computed tomography (CT) data, the second moments of area and moments of inertia at successive transverse slices along the rostrum were calculated for each of the species. Size-independent results tested the biomechanical benefits of material distribution within the rostra. The two spinosaur rostra were both digitally reconstructed from CT data and compared against all three crocodilians. Results show that African slender-snouted crocodile skulls are more resistant to bending than an equivalent sized gharial. The alligator has the highest resistances to bending and torsion of the crocodiles for its size and greater than that of the spinosaurs. The spinosaur rostra possess similar resistance to bending and torsion despite their different morphologies. When size is accounted for, *B. walkeri* performs mechanically differently from the gharial, contradicting previous studies whereas *Spinosaurus* does not. Biomechanical data support known feeding ecology for both African slender-snouted crocodile and alligator, and suggest that the spinosaurs were not obligate piscivores with diet being determined by individual animal size.

## Introduction

Extant crocodilian rostral morphology has often been used as an indicator of feeding ecology due to a link between head-shape and prey type or feeding behaviour [1]–[7]. These principles have been extended to various fossils forms with similar rostral morphologies in an attempt to determine diets [8]–[11]. Large, flattened skull morphologies tend to utilise lunge/ambush methods to capture food, with ‘death roll’ inertial feeding being used to break down terrestrial prey whilst narrower rostra often using slashing behaviours to capture fish [1]–[11]. Testing these correlations biomechanically has become important in attempting to understand not only extant crocodilians, but also reptilian feeding ecology in general [12]–[17]. Two distinct snout morphologies occur within archosaurs. Oreinirostral morphologies are high, tall domed snouts (as found in dinosaurs, pterosaurs, and many extinct archosaurs), and platyrostral morphologies are broad and flat snouts (common to most extant crocodilians and some extinct crurotarsans [18]). Most research shows that the oreinirostral snouts are stronger (or equivalent to platyrostral snouts) under tensile, compressive and rotational forces [18]. Crocodilians appear to have evolved a snout that was less tolerant to feeding related loads but potentially more suited to specialised hunting methods such as ambush [9] and hydrodynamic efficiency [5], [6].

Within platyrostral morphologies, there is a spectrum of morphological forms. At one extreme, the Gavialoidea (gharials and relatives) develop narrow and tubular longirostral snouts, whilst Alligatoroidea develop broad blunt snouts [19], [20]. The longirostral snout has long been associated with piscivory, with the gharial (*Gavialis gangeticus*) being the most highly derived and almost exclusively piscivorous [21], using rapid, swiping lateral strikes of the head to capture prey [22]. At the other extreme, the American alligator (*Alligator mississippiensis*) has the broadest snout of extant crocodilians, and mature individuals are able to feed on mammals (81.4% of the diet, with fish comprising 15.1%) and crush large turtles [23], [24].

*Mecistops cataphractus* (also known as *Crocodylus cataphractus*, commonly known as the African slender-snouted crocodile) is perhaps the most basal of extant crocodylid species [25], [26]. It lives in freshwater habitats in central and western Africa and possesses a longirostral snout with terminal rosette, bearing some resemblance to the gharial. Unlike gharials, the nasals are not separated from the premaxillae by the maxillae (similar to that of other extant crocodilians and spinosaur rostra), and the rostrum tapers from the posterior skull to the terminal rosette (contrary to the gharial rostrum, which is a fairly uniform width along the entire length from the orbits to the terminal rosette). The diet of *M. cataphractus* varies widely throughout the crocodiles’ range. Reports vary from exclusively piscivorous in some geographical areas, to a highly diverse diet including crabs, snakes, and even a small artiodactyl taken by a large individual [27].

The spinosaurids are a group of large theropod dinosaurs [28] that have been found in Africa, Asia, Europe and South America [29]–[32]. These “crocodile-mimic” dinosaurs possess an elongate, mediolaterally compressed ‘oreinirostral’ skull with a terminal rosette of subconical teeth, and posteriorly displaced internal and external nares [14]. The spinosaurid rostrum is distinct from that of other theropod dinosaurs and has been compared to that of modern crocodiles, especially that of the gharial. Such similarities in skull form have led to suggestions of piscivorous feeding behaviour in spinosaurs [14], [33]–[35]. Other evidence for piscivory includes a large claw on manual digit I in *B. walkeri* that may have functioned as a gaff for catching fish [35] and gastric acid etched *Lepidotes* fish scales in the rib cage of *B. walkeri* [32], [36]. Evidence suggests that spinosaurs were not exclusively piscivorous [26]. Juvenile *Iguanodon* bones were also found in the stomach region of *B. walkeri* [36], [37], and a South American spinosaur (likely *Irritator*) tooth has been found embedded within a pterosaur cervical vertebra [38].

Using a biomechanical approach, Rayfield et al. [33] tested the cranial biomechanics of *B. walkeri*, gharial and alligator specimens using finite element (FE) models. A hypothetical theropod (based on *Allosaurus*) was also modelled. Each of the models were loaded with equal bite forces (both bilateral and unilateral), and tensile and compressive stresses were calculated. The results showed that torsional stress was significantly higher than bending stress in the theropod and alligator, but there was no significant difference in the gharial and *B. walkeri* [33]. From this the authors inferred that *B. walkeri* and other spinosaurids were partially (if not completely) piscivorous. Therrien et al. [39] applied beam theory to the hemimandibles of extant species of monitor lizards and crocodiles as well as several theropods including *Suchomimus* (a spinosaur from North Africa). The ability of *Suchomimus* jaws to resist bending and torsion suggested that these animals also fed on fish and small terrestrial prey, using the anterior-most jaws to capture and manipulate prey.

**Figure 1:**
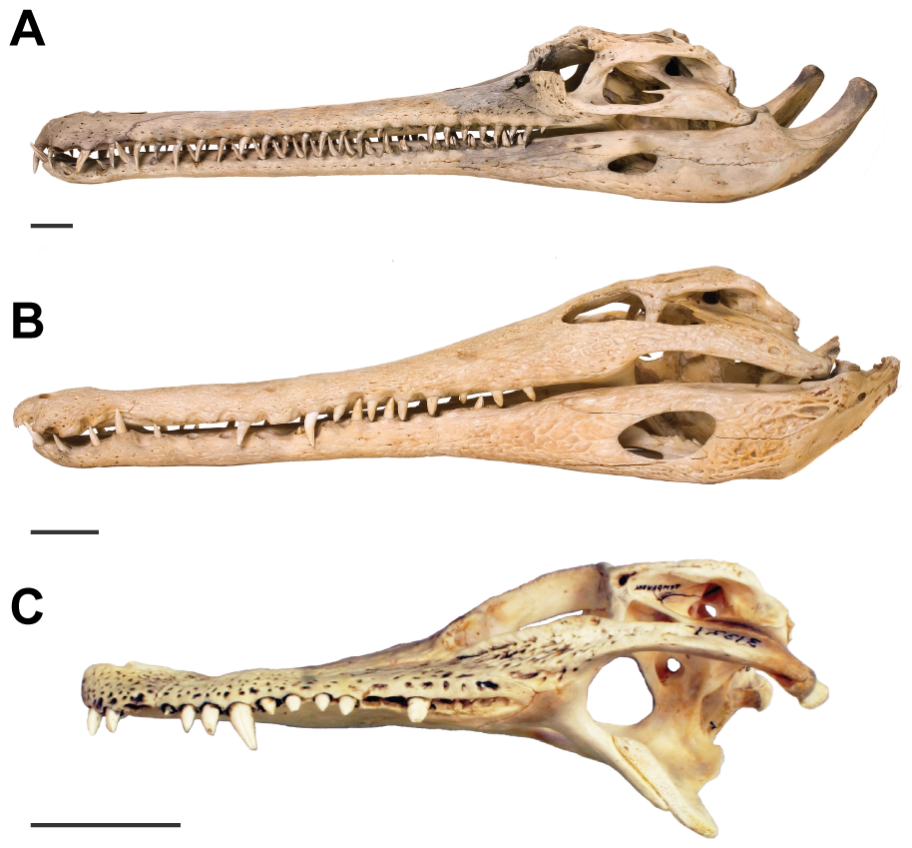
Species tested for second moments of area and moments of inertia. (A) *G. gangeticus* (gharial) – NHMUK 2005.1605 (specimen used here), (B) *M. cataphractus* – NHMUK 1924.5.10.1 (specimen used here), (C) *A. mississippiensis* (American alligator) for reference – Chicago Zoological Society 31321. Scale bars  =  5 cm.

**Figure 2:**
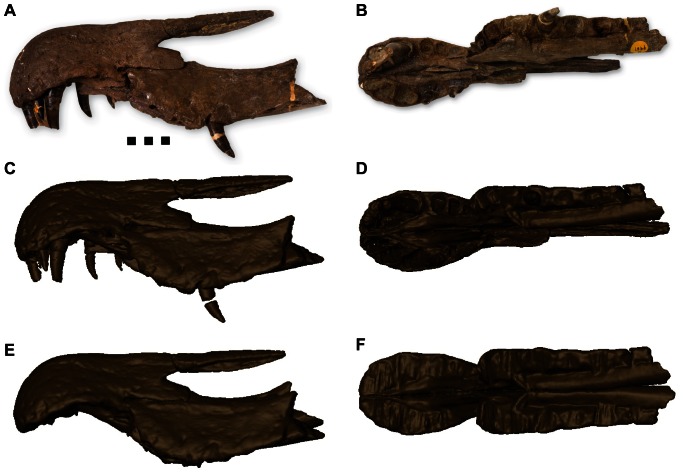
Lateral and ventral views of *Baryonyx walkeri* (NHMUK VP R9951) through the stages of digital preparation. (A) The original specimen in left lateral view, (B) the original specimen in ventral view, (C) the digitally prepared original in left lateral view, (D) the digitally prepared original in ventral view, (E) final specimen with teeth removed and alveoli levelled, (F) final specimen with teeth removed and alveoli levelled showing cloned right maxilla. See Video S1 and S2 for more detailed visualisations of the preparation and reconstruction. Scale bar  = 5 cm.

**Figure 3:**
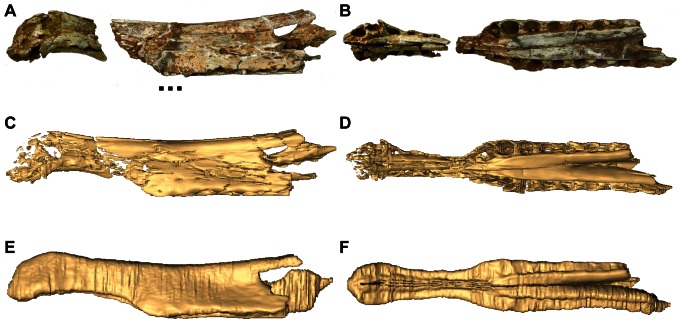
The digital preparation of *Spinosaurus* indet. **(NHMUK 16665) in lateral and ventral views**. The original specimen – lateral view (A), and ventral view (B). The digitally prepared specimen with no matrix – lateral view (C), and ventral view (D). The rostral reconstruction is based on other specimens of *Spinosaurus* (e.g. [28]) and the *B. walkeri* rostra - lateral view (E) and ventral view (F). Video S3 and S4 for more detailed visualisations of preparation and reconstruction. Scale bar  = 5 cm.

In the present study we supplement the computed tomography (CT) data used to create the FE-models of Rayfield et al. [33] with CT data from additional taxa. We use beam theory to determine the relative resistances to bending and torsion in the rostra and mandibles of three extant crocodilians (Figure 1) and rostra of two extinct spinosaurid dinosaurs (Figures 2 and 3). The aims of this study were (1) to test the comparative biomechanical properties of the rostra of *M. cataphractus*, gharial and American alligator; (2) to test the biomechanical properties of spinosaur rostra (*Spinosaurus indet.* cf. *Spinosaurus aegyptiacus* and *B. walkeri*) relative to all three crocodilians; and (3) to gain insight into the functional mechanics of piscivorous archosaurs. The results of the study will help understand the relationship between form, biomechanical properties and feeding ecology within crocodylians, and has the potential to be extended to extinct archosaurs. This may help further understand the structural integrity of the spinosaur rostrum, and whether spinosaurs converged mechanically upon a gharial-like piscivorous snout, or maintained a more generalist rostrum.

## Methods

### Imaging

Computed tomography (CT) data was obtained for each of the five species. The American alligator data (from a juvenile, Texas Memorial Museum, University of Texas at Austin, Austin, Texas, USA (TMM) TMM m-983, skull length 21.7 cm) were obtained from ‘Digital Atlas of the Alligator’ (available in [40]), scanned at a slice thickness of 480μm. The gharial skull (The Natural History Museum, London, United Kingdom (NHMUK) NHMUK 2005.1605 – a very large adult with a skull total length of 86 cm) was scanned at the Royal Veterinary College, Potters Bar, UK 120 kV, 200 mA, Field of View (FOV) = 320×320 pixels, 5 mm slice thickness, and *M. cataphractus* (NHMUK 1924.5.10.1 – an adult skull of 62 cm) 120k V, 150 mA, FOV  = 280×280 pixels, 5 mm slice thickness. *B. walkeri* (NHMUK PV R9951 – probably subadult) co-joined premaxillae and left maxilla were scanned at University of Ohio O’Bleness Memorial Hospital; FOV  = 151×151 mm (premaxilla); 188×188 mm (maxilla) at a slice thickness of 1.25 mm. The *Spinosaurus* rostra (NHMUK 16665) was scanned at Royal Veterinary College, Potters Bar, UK, 120 kV, 150 mA, FOV  = 200×200 mm with a slice thickness of 5 mm.

### Digital preparation of spinosaurs

The CT scans of *B. walkeri* and *Spinosaurus* were visualised using *AVIZO* 6.1.1 (VSG SAS, Bordeaux, France). Using the labelling function, the matrix was virtually removed from the scans, leaving only bone. The *B. walkeri* rostrum is missing the anterior portion of the right maxilla (Figure 2, Video S1), so this was reproduced by creating a mirror clone of the equivalent portion of left maxilla (Figure 2, Video S2). The *Spinosaurus* rostrum is heavily damaged, and the premaxilla is especially fragmented (Figure 3, Video S3). To compensate for this damage, the skull was digitally reconstructed (Figure 3, Video S4) as accurately as possible, using the existing material and images from other known specimens (e.g. Museo Civico di Storia Naturale di Milano, Milan, Italy (MSNM) MSNM V4047, [28]).

### Application of Beam Theory

**Figure 4:**
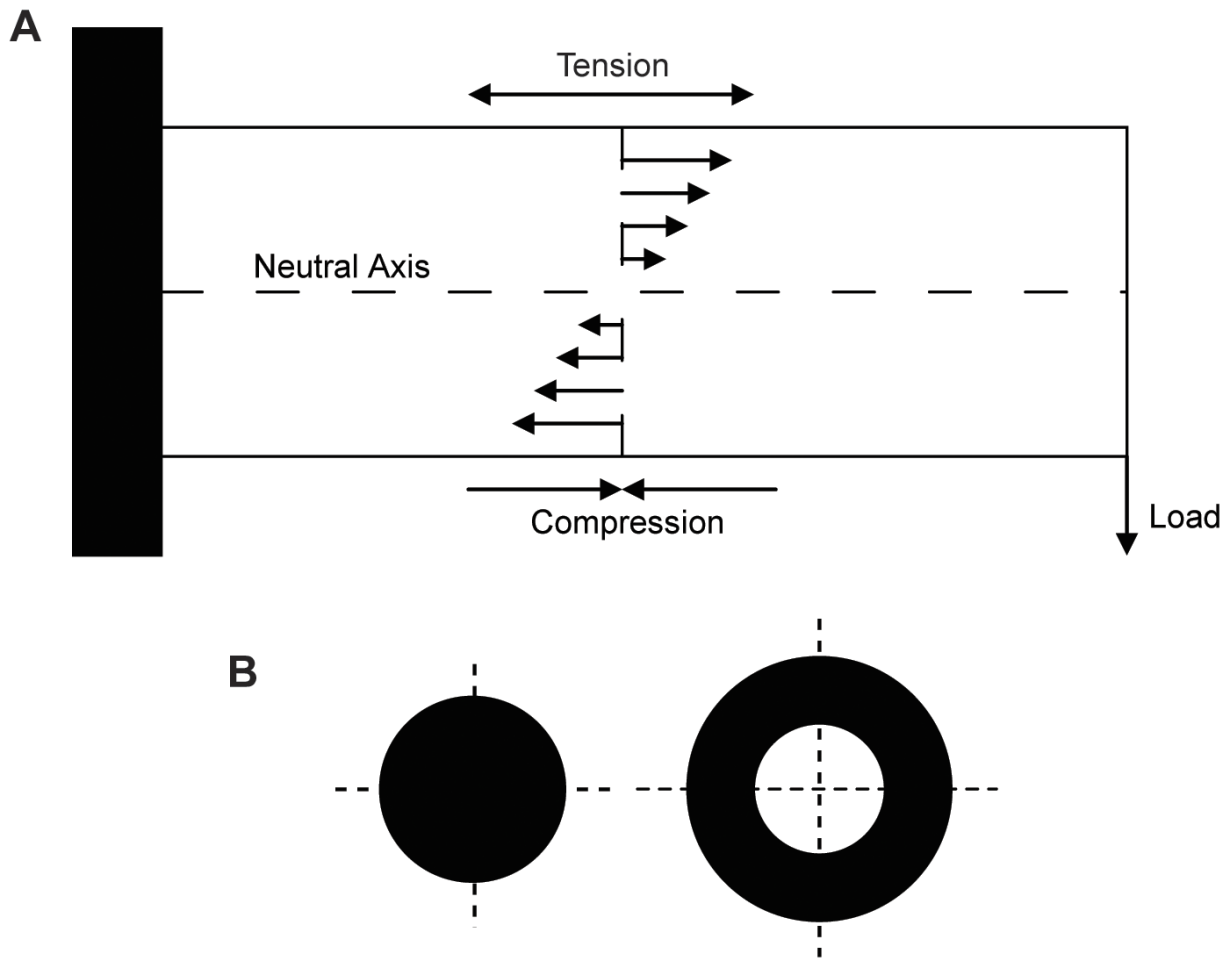
Simple illustrations of beam theory . (A) When a load is applied to a beam with one fixed end (a cantilever beam), the effect of the beam is a deflection in the direction of the force. This results in the most extreme tension on one side of the beam, and the most extreme tension on the opposite side. In the middle, there is a point where there is no tension or compression, called the neutral axis. B) Two circular cross sections of equal cortical area (black). Beam theory states the solid tube (hollow circle) will have higher resistance to bending and torsion than the solid circle due to the material being distributed further from any neutral axis.

Beam theory is an engineering method that allows for the study of simple cantilever beams, those fixed at one end. A number of studies have approximated the rostra of tetrapods as cantilever beam in order to calculate rostral resistance to dorsoventral and mediolateral bending, and torsion about the longitudinal axis [3], [41]–[44] . In these instances it is assumed that rostra meet the criteria for deflection of a cantilever beam under load, namely that load is applied to the free end of the beam, the structure is longer than it is thick or wide, and material properties are constant (isotropic and heterogeneous) along the length of the beam. It is assumed here (as has been the case in previous studies) that these criteria are met; however, the implications of such assumptions are considered further in the discussion. Here we calculate the second moment of area and the polar moment of inertia of successive slices through the rostra of our selected taxa, from the tip of the snout to just anterior to the orbital margin. Using this method, it is possible to quantify how the distribution of bony material within a rostral cross-section affects its resistance to bending and torsion. For example, if two cylinders with the same amount of material are compared, the one with the greatest overall radius will withstand larger forces before buckling (Figure 4).

Second moments of area are calculated using the equation:
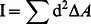
where I  =  second moment of area, d  =  distance from neutral or centroidal axis (where there is no compressive or tensile load), and ΔA  =  strip of material within the structure. If I is multiplied by the Young’s modulus of the material the result is the flexural stiffness of the structure. The sum of the second moments of area in the dorsoventral (Ix) and mediolateral (Iy) directions give the polar moment of inertia (J). When J is multiplied by the shear modulus, the result is the torsional stiffness of the structure, also known as the resistance to torsion. Where structures share the same material properties, the relative values of I and J indicate relative flexural and torsional stiffness.

#### (1) Testing comparative biomechanical properties of the crocodilian rostrum

**Figure 5:**
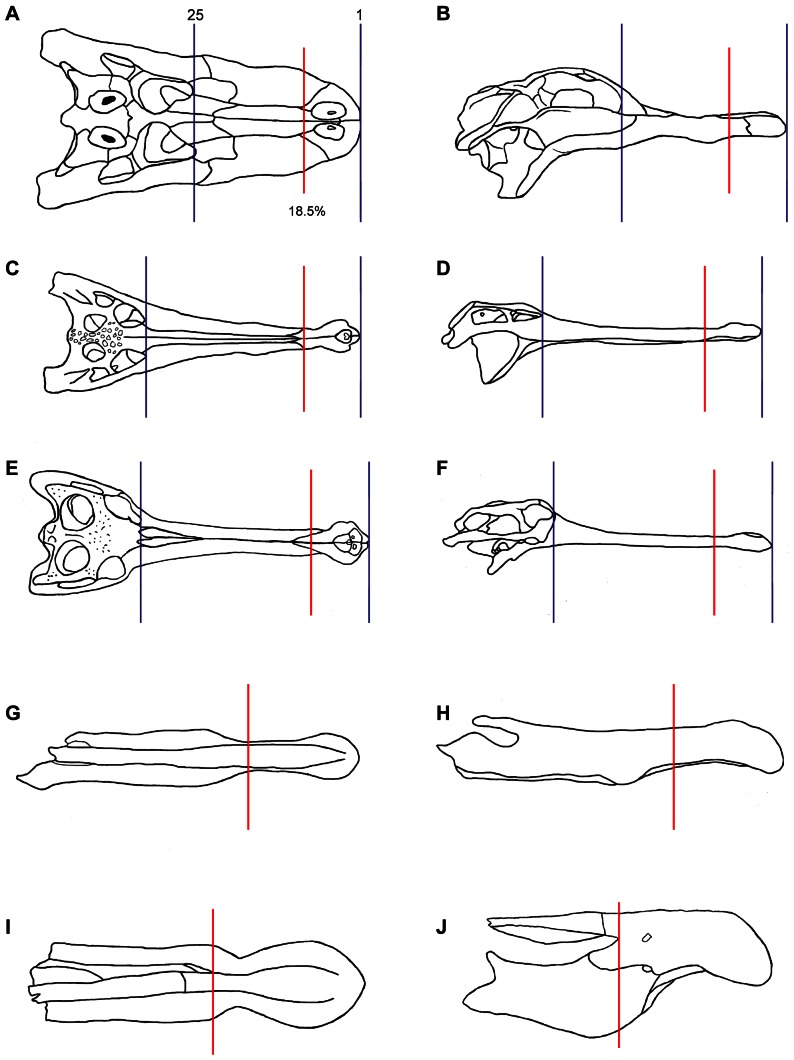
Dorsal and lateral views of skulls/reconstructed rostra of the species tested showing slice locations. (A) *A. mississippiensis*, (B) *G. gangeticus*, (C) *M. cataphractus*, (D) *Spinosaurus* indet. and (E) *B. walkeri.* All skulls have had their teeth removed and alveoli leveled. Blue lines indicate first (1) and last (25) slices of the crocodilian study, red lines mark on the spinosaurs (or equivalent for the crocodilians): 1^st^ slice located at the rostral tip; 8th slice located at 18.5% of total rostral length.

For each of the species (*A. mississippiensis*, *G. gangeticus*, *M. cataphractus*), we analysed Ix, Iy and J for 25 equally spaced CT slices from the anterior portion of the premaxillae to the slice immediately anterior to the orbits (Figure 5). CT images were first converted to black and white images. Teeth influence second moment calculations by changing the apparent area of cortical bone in any cross section, which can lead to an increase in I and J values in any given CT slice. Thus, to standardize the effects of teeth and their alveoli, all teeth were removed and alveoli filled to the level of the alveolar socket to create a closed section [45].

#### (2) Comparing spinosaur and crocodilian rostra

Both spinosaurs have only a small portion of the rostrum intact in the specimens. Only the premaxillae and the anterior portion of the maxilla encircling the anterior border of the external naris are preserved and the nasals are missing in both taxa. We estimated the total length of the skull (rostral tip of premaxilla to posterior edge of quadrate) for *B. walkeri* from the reconstruction in [31] and for *S*. cf. *S. aegyptiacus* from the composite reconstruction of [28]. The lack of nasals in *B. walkeri* meant we were limited to using useful comparative CT slice data from only the anterior portion of the *B. walkeri* skull, equivalent to 18.5% of total skull length (red line, Figure 5I, J). We therefore calculated Ix, Iy and J values for eight equally spaced CT slices from the anterior 18.5% of total skull length for each crocodilian and spinosaur. Because the spinosaur CT data was restored, and for *B. walkeri*, reflected, the spinosaur CT cross sections were created from thresholded labels, produced after digital preparation and restoration in *AVIZO*. Again, the teeth were removed and alveoli were filled to prevent bias.

### Data acquisition and manipulation

The prepared image files were opened in *ImageJ*, free and open source software downloadable from http://rsb.info.nih.gov/ij/ [46]. *MomentMacroJ* v1.3, a free macro available from http://www.hopkinsmedicine.org/FAE/MMacro.htm [47], was used to calculate Ix and Iy. *MomentMacro* calculates second moment of area for all pixels within a user-defined greyscale threshold (rostral bone in this case). Ix and Iy were summed to calculate J.

To then correct for size discrepancy between our chosen specimens, we used data manipulation tools in *AVIZO* to scale all CT data to the length of the skull of *G. gangeticus*. The aspect ratio of each slice was maintained. This resulted in a modified scan dataset representing the three crocodilian and two spinosaur skulls scaled to equal length dimensions (rostral tip of premaxilla to posterior edge of quadrate, as before).

### Tests

To test if any of the crocodilian or dinosaurian species are similar in their resistances to bending or torsion, paired comparisons of Ix, Iy and J were carried out between the crocodilian rostra and the dinosaur rostra. For all crocodilians, no combinations of pairings both passed normality tests, so the data were tested with a non-parametric Wilcoxon paired test (in *STATISTICA* v.6, StatSoft Inc. 2003), to test for similarities in resistances to bending and torsion along the rostra lengths at equivalent locations. Only the tests for both absolute and size-corrected data with the *B. walkeri* Iy were not normal and pairings containing these data were tested as before with Wilcoxon paired tests. The other pairings were tested using a two-tailed T test. Due to the number of tests carried out, the p values for significance were adjusted for each test using a Šidàk correction [48]. This is the equation from which the Bonferroni correction is derived and is more accurate. The new probability for each test is calculated by:
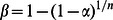
where α is the original probability (in this case 0.05) and n is the number of tests carried out.

## Results

### Crocodilians

**Figure 6:**
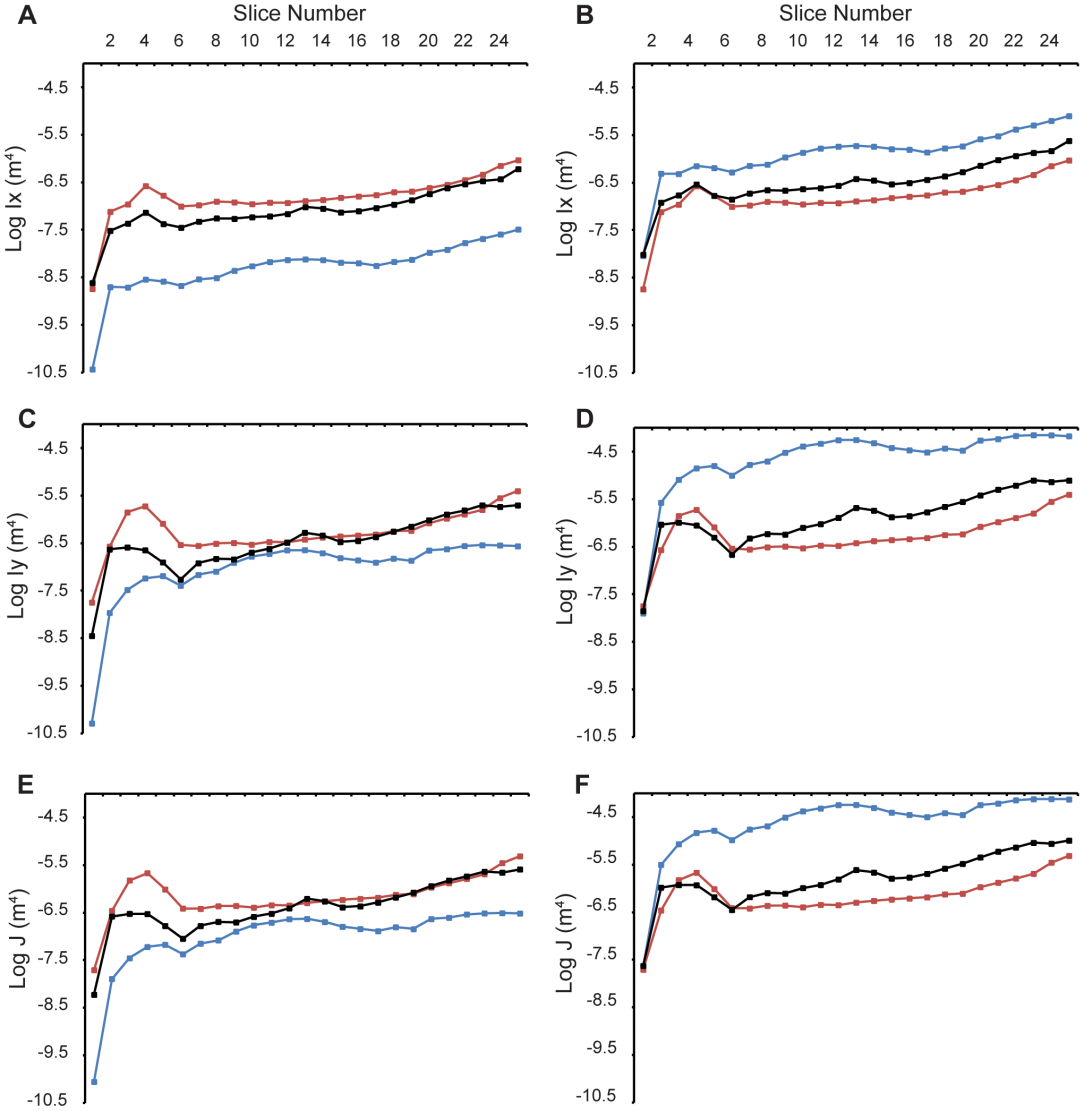
Log of absolute and size-corrected second moments of area and moments of inertia for crocodilians. (A) log absolute Ix , (B) log size-corrected Ix (C) log absolute Iy , (D) log size-corrected Iy, (E) log absolute J , (F) log size-corrected J. Blue  =  alligator, red  =  gharial, black  =  *M. cataphractus*. Squares  =  upper jaw.

Resistance to dorsoventral bending (Ix) for raw (Table S1) and size-corrected (Table S2) data shows the same trends for all species; all taxa show a minor peak at slice 4 (16% of the rostra) and then a slight increase in Ix values towards the posterior of the rostrum (Figures 6a and 6b). The raw values for the gharial are generally highest, with the alligator approximately 100 times smaller (Figure 6a), reflecting the actual size of the specimens. When size-corrected, the order is flipped with the alligator having the highest Ix values by a factor of 10, whilst the gharial has the lowest values (Figure 6b).

Values of Iy, mediolateral resistance, are greater than those of Ix, dorsoventral bending (Figures 6c and 6d). All species exhibit peaks between slices 2 and 6 (8 and 24% of skull length) before exhibiting steady rises to the posterior of the rostra. Unlike the resistances to dorsoventral bending, the mediolateral results between the gharial and *M. cataphractus* are much similar, with *M. cataphractus* having several locations where the resistances to bending are greater than that of the gharial. When size-corrected, the alligator again has the largest Iy values by a factor of 10 to 100 (Figure 6d). The gharial has greater Ix values than *M. cataphractus* for the anterior 24% of the skull, but further posterior *M. cataphractus* exhibits greater Iy values.

As the J values (resistance to torsion) are the sum of Ix and Iy, trends in the magnitude of J tend to follow those of the largest resistances, in this case the Iy values (Figure 6e). This is also true for the size-corrected values (Figure 6f). Hence when size-corrected, material distribution in the alligator reflects the greatest resistance to torsion.

After carrying out pair tests, the raw data for the gharial Iy vs. *M. cataphractus* Iy are not significantly different, as are gharial J vs. *M. cataphractus* J (although only after the Šidàk correction for multiple tests). All other raw data pairings for Ix, Iy and J are not significantly different showing no statistical differences between the taxa (Table 1). When corrected for size, all data pairings are significantly different (Table 1).

**Table 1:** Wilcoxon tests for the upper jaw pairings of the crocodilian species for both size-corrected data and residuals.

Taxon 1	Taxon 2	Raw		Size-corrected	
		z	p value	z	p value
Ix					
Alligator	Gharial	4.37	<0.001	4.37	<0.001
Alligator	*M. cataphractus*	4.37	<0.001	4.35	<0.001
Gharial	*M. cataphractus*	4.35	<0.001	4.37	<0.001
Iy					
Alligator	Gharial	4.37	<0.001	4.35	<0.001
Alligator	*M. cataphractus*	4.37	<0.001	4.35	<0.001
Gharial	*M. cataphractus*	1.76	<0.001	3.57	<0.001
J					
Alligator	Gharial	4.37	<0.001	4.37	<0.001
Alligator	*M. cataphractus*	4.37	<0.001	4.35	<0.001
Gharial	*M. cataphractus*	2.70	0.00685*	3.78	<0.001

Results that shift from significant to non significant after Šidàk test are marked with an asterisk (*).

### Spinosaurs

**Figure 7:**
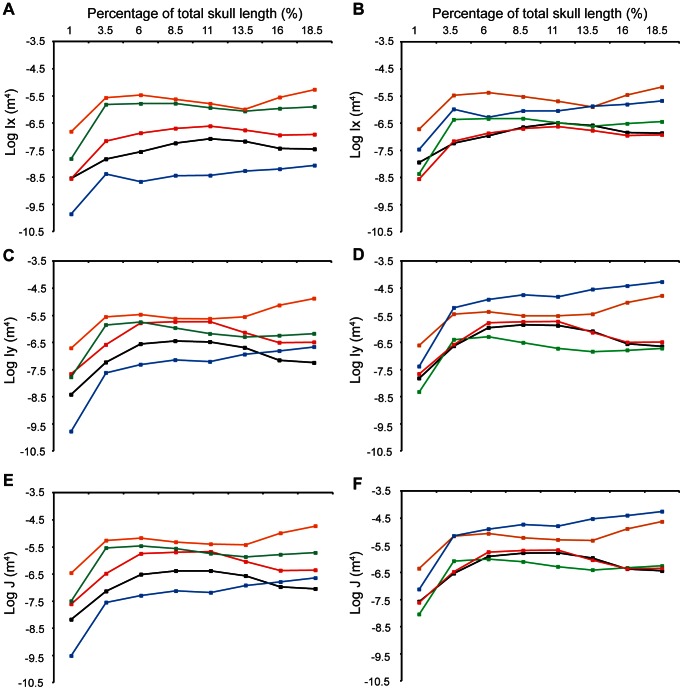
Log of absolute and log of size-corrected second moments of area and moments of inertia for crocodilians and spinosaurid rostra. (A) log absolute Ix , (B) log size-corrected Ix (C) log absolute Iy , (D) log size-corrected Iy, (E) log absolute J , (F) log size-corrected J. Blue  =  alligator, red  =  gharial, black  =  *M. cataphractus*, green  =  *Spinosaurus*, orange  =  *B. walkeri*.

For the raw Ix data, both the spinosaurs have similar values and have resistances to dorsoventral bending that are higher than all of the crocodilian species (Figure 7a, Table S3). When size-corrected, the *B. walkeri* resistances to dorsoventral bending remain higher than all other species, but the *Spinosaurus* falls between the alligator and the gharial (Figure 7b, Table S4).

When raw data are considered, *B. walkeri* still has greater Iy (mediolateral bending resistance) values than all other taxa, yet *Spinosaurus* has similar and in some cases lower resistance than the large gharial skull used in this study (Figure 7c). For both spinosaurs, values of Iy are closer in value to those of the crocodile species than are values for Ix (Figure 7c). The size-corrected values show that an alligator of the same skull length as a spinosaur has a greater Iy values and therefore a greater resistance to mediolateral bending. *Spinosaurus* values are lower than those for all crocodilians, whilst the *B. walkeri* resistance to bending falls between the alligator and the gharial (Figure 7d).

The raw data for resistance to torsion show higher values for the spinosaurs compared to the crocodilian species, except at 11% along the jaw for the *Spinosaurus* when compared to the gharial. The higher values of J are due to the much higher resistance to dorsoventral bending in spinosaurs compared to crocodilians (Figure 7e). When corrected for size, the *Spinosaurus* rostra performs equal to, or slightly worse than both the gharial and *M. cataphractus*. *B. walkeri*, however, is intermediate between the alligator and the other crocodilians (Figure 7f).

After Šidàk correction only the *Spinosaurus* Ix vs gharial Ix, alligator Ix and *M. cataphractus* Ix are significantly different. For size corrected data all pairing p-values become non-significant after correcting for multiple tests (Table 2).

**Table 2:** Two tailed t-tests and Mann Whitney tests between the spinosaurids and the crocodilian species for both size-corrected data and residuals.

Taxon 1	Taxon 2	Raw		Size-corrected	
Ix		t	p value	t	p value
*Spinosaurus*	*B. walkeri*	–2.76	0.0281*	4.04	0.00494*
*Spinosaurus*	Gharial	5.73	<0.001	–4.23	0.00387*
*Spinosaurus*	Alligator	6.03	<0.001	3.42	0.0111*
*Spinosaurus*	*M. cataphractus*	5.90	<0.001	–2.92	0.0222*
*B. walkeri*	Gharial	4.09	0.00463	4.14	0.00437*
*B. walkeri*	Alligator	4.31	0.00354	3.46	0.0105*
*B. walkeri*	*M. cataphractus*	4.23	0.00389	4.06	0.00481*
Iy					
*Spinosaurus*	*B. walkeri*	z = 2.52	0.0117*	z = 2.52	0.0117*
*Spinosaurus*	Gharial	0.139	0.893	2.51	0.0402*
*Spinosaurus*	Alligator	–3.68	0.00781*	3.37	0.012*
*Spinosaurus*	*M. cataphractus*	–3.65	0.00816*	2.42	0.0460*
*B. walkeri*	Gharial	z = 2.52	0.0117*	z = 2.52	0.0117*
*B. walkeri*	Alligator	z = 2.52	0.0117*	z = 2.38	0.0173*
*B. walkeri*	*M. cataphractus*	z = 2.52	0.0117*	z = 2.52	0.0117*
J					
*Spinosaurus*	*B. walkeri*	2.50	0.0408*	3.24	0.0143*
*Spinosaurus*	Gharial	–2.93	0.0220*	1.71	0.130
*Spinosaurus*	Alligator	–5.05	0.00149*	3.37	0.0119*
*Spinosaurus*	*M. cataphractus*	–5.02	0.00152*	1.34	0.222
*B. walkeri*	Gharial	–2.81	0.0261*	–2.94	0.0217*
*B. walkeri*	Alligator	–3.43	0.0110*	3.10	0.0172*
*B. walkeri*	*M. cataphractus*	–3.30	0.0130*	–3.02	0.0193*

Results that shift from significant to non significant after Šidàk test are marked with an asterisk (*).

## Discussion

Results for the raw uncorrected data tend to reflect the differences in skull sizes. The alligator skull was the smallest at 21.7 cm length, *M. cataphractus* measured 62 cm and the gharial was the longest skull at 86 cm. The size order is reflected in the relative resistance to bending and torsion in the absolute raw data. Even the platyrostral alligator skull has lower Iy values than the tubular gharial and *M. cataphractus*, due to its small size. Thus, inferences for the functional morphology of crocodilians and spinosaurs are best interpreted from the size-corrected data. The gharial specimen represents an extremely large mature individual, and the *M. cataphractus* skull is interpreted as belonging to an adult based on its large size. In a comparison of ontogenetic trajectories in four crocodilian taxa, the gharial and *M. cataphractus* had the lowest covariation between rostrum shape and size (when considered alongside *Tomistoma* and *Crocodylus acutus*) [49]. Hence the size-corrected *M. cataphractus* may be a reasonable interpretation of the outline shape of a 80-plus centimetre long specimen. *A. missippiensis*, however, shows snout elongation and narrowing through ontogeny [50] yet scaling of bite force to head and jaw length reveals positive allometry [51]. These data suggest that our scaling of a sub-adult alligator to very large adult size probably does not fully reflect the morphological changes that occur during ontogeny. One further issue is that the length-scaling method, although retaining the aspect ratio of the transverse slices, will not account for increases in cortical bone thickness and increased ornamentation that may occur in older, larger specimens. These issues should be borne in mind. However, our analysis will still capture the main differences in morphology between the crocodilian taxa.

The wider, more robust alligator skull possesses higher Iy and J values that increase to the broadest part of the skull, whilst the gharial results reflect its regular, tubular rostrum. *M. cataphractus* results show fairly regular values (close to that of the gharial) for the anterior 40% of the snout. By standardizing the datasets, it can be inferred that the alligator has the most biomechanically efficient rostra for resisting bending and torsion, the gharial the least, and *M. cataphractus* intermediate between the two extremes, but closer to the gharial than the alligator.

Busbey [9] recognised three behaviours that have the potential to exert the greatest stresses on the rostrum of platyrostral crocodilians. These were (1) biting down on prey in the mouth; (2) rolling; (3) pitching (up/down) or yawing (side-to-side) of the head. Biting and pitching generate dorsoventral bending and stresses along the dorsal and ventral aspects of the rostrum. Rolling generates axial torsion along the rostrum, whilst yawing results in mediolateral bending. As well as being an adaptation for feeding behaviour, rostral shape may be influenced by mechanical constraints to minimize feeding-induced stress, developmental and phylogenetic constraints, and hydrodynamic demands [2], [5], [6], [9], [13]. Our study shows that for similar sized specimens, alligators have a greater second moment of area and moment of inertia than gharials and *M. cataphractus* along the length of their skull. Our results are consistent with those of Busbey [9] who found the largest second moment of area in *A. mississippiensis* compared to other crocodilians of similar skull length, including *M. cataphractus*. The higher resistance to torsional loading in alligator may be related to their feeding strategy. The alligator is well known for its twist feeding strategy, the so called “death roll”, of which even young alligators are capable [52], [53]. Such spinning behaviour reduces large or tough prey into manageable pieces, and imparts a shear force to enable dismemberment or breakdown of the prey item. In turn, the rostrum is subject to large torsional loading and our results are consistent with resistance to such loads. The alligator in our study has a skull length of 21.7 cm, so the total length of the animal was approximately 140 cm−160 cm [54]. For an individual of this size, the primary food source varies depending on location, from fish to birds and small mammals, although it is possible that medium-sized mammals and turtles may be taken [22], [23]. Hence, twist feeding is a possibility for an animal of this size. A broader comparison using finite element modelling of the mechanical performance of *A. mississippiensis* and other short, broad crocodilian taxa suggests that the platyrostral morphology of alligator is far from optimal at torsion resistance [5] but performs reasonably well in comparison to all extant crocodilian species [6]. Our results support the suggestion that alligator cranial morphology may represent a compromise between feeding behaviour and hydrodynamic efficiency [5], [6].

The gharial uses a slashing motion through the water to stun and capture fish [21]. Its longirostrine morphology leads to greater angular acceleration and therefore greater speed at the end of the rostrum [5], [22], and a narrow tubular morphology reduces surface drag [6]. Gharials are morphologically distinct [6], [49] and have a diet consisting almost entirely of fish [22]. Prey capture may be expected to impart mediolateral and dorsoventral loads on the rostrum during prey capture and inertial feeding. This is reflected in the tubular rostral morphology. The large size of our gharial specimen leads to large second moment and moment of inertia values. However, when size-corrected, the gharial is the poorest performing of the three crocodilian taxa.

In comparison *M. cataphractus* performs slightly better than the gharial when size-corrected. Evidence of prey choice and feeding behaviour in this latter taxon is sparse. All six of the individuals from Lake Divangui (Gabon) were between 200 cm and 235 cm in total length (smaller than the individual used in this test) and contained exclusively fish in their stomachs [27]. However, a larger individual from another region of Gabon had the remains of a small artiodactyl in its stomach [27]. In the absence of known methods of prey capture, it appears that *M. cataphractus* prey selection may, as in alligators, be determined by the size of the individual, which in turn affects the size of the rostrum and overall absolute resistance to bending and torsion. The slight increase in bending and torsion resistance in the rostrum of *M. cataphractus* may reflect this fact.

Despite the differences in the size and morphology of the tested regions between the *Spinosaurus* (estimate skull length 117.6 cm; longer, more gracile and a small terminal rosette relative to length) and *B. walkeri* rostra (97.1 cm estimated length; therefore shorter, relatively more robust with a larger terminal rosette), both spinosaur rostra perform in a similar manner, and due to their large size absolutely outperform all crocodilian taxa. This points to spinosaurid feeding methods potentially being very similar, at least between these two species. When size is accounted for, the larger spinosaur, *Spinosaurus*, performs worse than *B. walkeri*. Relative to the crocodilians, the spinosaurs generally both have higher absolute resistances to bending and torsion. In terms of absolute resistance to torsion and mediolateral bending, the large gharial is the closest functional analogue of the living crocodilians studied here. However, when the effects of size are removed, the pattern changes somewhat. The large dorsoventral second moment values for *B. walkeri* are consistent with previous studies documenting greater dorsoventral bending resistance in orienirostral taxa such as the extinct crocodylomorph *Sebecus ichaeorhinus*, and the extant caiman *Melanosuchus niger* and *Paleosuchus palpebrosus* [2], [5], [9]. This is also true of the poor performance of both spinosaurs in mediolateral bending and torsion resistance. Interestingly, the rostral shape of *Spinosaurus* is less resistant to dorsoventral bending than an alligator of similar size, and performs worse than all crocodilians in mediolateral bending, including tubular gharial morphotypes. The trends in second moment and torsional resistance are similar along the rostrum, yet *B. walkeri* rostra are more robust. However, this study was only able to compare performances of the anterior rostrum and the results should be considered in this context.

These results differ from those found by Rayfield et al. [33], which suggested that the *B. walkeri* and gharial rostra are functionally convergent in terms of their resistance to bending and torsional feeding loads [33]. Only the size-corrected resistances to torsion of *Spinosaurus* are similar to those of the gharial.

Consideration of the functional anatomy of spinosaurs in a further study using second moments of area and moments of inertia attempted to understand theropod feeding[39]. Based on the dentary results, similarities to Orinoco crocodiles (*Crocodylus intermedius*), and length of the mandibular symphysis, the authors concluded that the spinosaurs probably fed on smaller prey, capturing them in their rosette of teeth and holding the prey or shaking their heads dorsoventrally, because their skulls were not very resistant to mediolateral bending [39], [55]. Here we find the same trend in the rostrum: the values obtained for *Suchomimus* dentaries in this previous study [39] are very similar to those calculated for the rostra of the spinosaurs in this study. Spinosaurs possess deep rooted teeth and near vertical-sided teeth rows, ideal for resisting large dorsoventrally orientated biting forces and dissipation of forces through the skull [55]. Calculations of bite force in *Suchomimus* [39] suggest that the bite may have been comparable to an alligator with a mandibular length of 50 cm suggesting that spinosaurs were capable of capturing terrestrial prey [39].

The results of this study must be taken in the context of the assumptions of beam theory, concerning the shape, loading regime and homogenous material composition of the rostrum. The results also assume that second moment of area and moment of inertia are useful proxies for bone strength and resistance to loads. Calculation of flexural and torsional stiffness rely on multiplication of I and J values by the Young’s modulus and shear modulus respectively. For the purpose of this study we have assumed that crocodilians and spinosaur theropod dinosaurs possess equivalent stiffness and shear values, and hence can be compared directly without consideration of potential differences in material properties. We will never know the exact material properties of extinct animal bone; however studies have shown that many taxonomically distinct vertebrates have similar moduli [56], and indeed there are similarities in the cranial material properties of crocodilian and mammalian bone [57].

## Conclusion

It appears that the spinosaur theropod dinosaurs studied here achieved superiority in resistance to bending and torsion over representative crocodilians by nature of their large size. When size is corrected for, *Spinosaurus* performs relatively poorly compared to the other taxa. In comparison, *B. walkeri* performs surprisingly well, its oreinirostral morphology conferring greater resistance to dorsoventral bending and torsion than *Spinosaurus* and the gharial, to which *B. walkeri* has been compared in the past. Whether influenced by hydrodynamic or feeding related constraints, a combination of both, or other factors, the size-corrected alligator rostrum is well-equipped to deal with mediolateral and torsional loads, compared to our other study taxa. Our results only consider the portion of the skull anterior to the external naris, and a consideration of a larger portion of the rostrum is desired before a more complete understanding of rostral function can be obtained.

In conclusion, the unusual rostral morphology of spinosaurs conferred some advantage in dorsoventral bending resistance, particularly in *B. walkeri*, yet both species studied here were poorly equipped to resist mediolateral and torsional loads. *Spinosaurus* represents one of the biggest, if not the biggest theropod dinosaur [58], yet scaled to the size of an alligator, gharial or slender-snouted crocodilian, it performs poorly, especially in resistance to torsion. For a taxon such as *Spinosaurus*, the ability to feed on larger, struggling prey was not conferred by the possession of a snout that was relatively well equipped to deal with associated feeding loads, but may have been achieved by simple size-related advantages.

## Supporting Information

Table S1 **Resistances to bending and torsion in absolute values for crocodilian upper jaws.** All values are metres ×10^−07^.
(DOC)

Table S2 **Resistances to bending and torsion in size-corrected, crocodilian upper jaws.** All values are metres ×10^−07^.
(DOC)

Table S3 **Absolute values for resistances to bending and torsion in dinosaurian and crocodilian rostra.** All values are metres ×10^−07^.
(DOC)

Table S4 **Resistances to bending and torsion in size-corrected dinosaurian and crocodilian rostra.** All values are metres ×10^−07^.
(DOC)

Video S1 **The original** ***Baryonyx walkeri*** **specimen digitally prepared from the CT data**. The broken rostromedial processes of the maxillae can be seen as a bone shard extending anteriorly from the premaxilla-maxilla suture.
(WMV)

Video S2 **The final** ***Baryonyx walkeri*** **specimen**. The right maxilla is cloned and mirrored to the left side, teeth removed and alveoli levelled. The expected positions of the rostromedial processes can be seen. The broken portion of the premaxilla above the external nares was not corrected as it did not affect the area being studied.
(WMV)

Video S3 **The digital prepared specimen of** ***Spinosaurus indet.*** The highly fragmented and distorted nature of the specimen can be seen.
(WMV)

Video S4 **The rostral reconstruction of** ***Spinosaurus indet.*** This was based on the existing material, other specimens of *Spinosaurus* (e.g. [28]) and the *B. walkeri* rostra.
(WMV)
