# Supplementary material for: Feeding Mechanics in Spinosaurid Theropods and Extant Crocodilians
Source: PLoS One. 2013 May 28;8(5):e65295. doi: 10.1371/journal.pone.0065295 (PMC3665537; doi:10.1371/journal.pone.0065295)
Supplement: Table S1 — Resistances to bending and torsion in absolute values for crocodilian upper jaws. All values are metres ×10−07. (DOC) [file pone.0065295.s001.doc]

**Table S1. Resistances to bending and torsion in absolute values for crocodilian rostra.** All values are metres x10^-07^.

| Slice | Alligator | | | Gharial | | | *M. cataphractus* | | |
| --- | --- | --- | --- | --- | --- | --- | --- | --- | --- |
|  | Ix | Iy | J | Ix | Iy | J | Ix | Iy | J |
| 1 | 0.000369 | 0.000512 | 0.000881 | 0.0181 | 0.180 | 0.200 | 0.0243 | 0.0355 | 0.0598 |
| 2 | 0.0198 | 0.109 | 0.129 | 0.762 | 2.71 | 3.47 | 0.304 | 2.35 | 2.65 |
| 3 | 0.0195 | 0.331 | 0.350 | 1.09 | 14.1 | 15.2 | 0.431 | 2.58 | 3.02 |
| 4 | 0.0286 | 0.576 | 0.605 | 2.66 | 19.0 | 21.7 | 0.733 | 2.25 | 2.98 |
| 5 | 0.0260 | 0.648 | 0.674 | 1.67 | 8.15 | 9.82 | 0.422 | 1.25 | 1.67 |
| 6 | 0.0211 | 0.405 | 0.426 | 0.984 | 2.91 | 3.90 | 0.353 | 0.546 | 0.899 |
| 7 | 0.0290 | 0.683 | 0.712 | 1.05 | 2.79 | 3.84 | 0.474 | 1.20 | 1.68 |
| 8 | 0.0308 | 0.803 | 0.834 | 1.25 | 3.10 | 4.35 | 0.555 | 1.49 | 2.04 |
| 9 | 0.0438 | 1.23 | 1.27 | 1.21 | 3.22 | 4.43 | 0.543 | 1.45 | 1.99 |
| 10 | 0.0547 | 1.66 | 1.72 | 1.11 | 2.96 | 4.06 | 0.585 | 2.01 | 2.60 |
| 11 | 0.0672 | 1.90 | 1.97 | 1.19 | 3.37 | 4.56 | 0.612 | 2.41 | 3.02 |
| 12 | 0.0737 | 2.25 | 2.33 | 1.17 | 3.33 | 4.50 | 0.685 | 3.25 | 3.94 |
| 13 | 0.0762 | 2.28 | 2.35 | 1.28 | 3.80 | 5.08 | 0.955 | 5.27 | 6.23 |
| 14 | 0.0736 | 1.95 | 2.03 | 1.35 | 4.19 | 5.54 | 0.886 | 4.61 | 5.50 |
| 15 | 0.0651 | 1.55 | 1.61 | 1.51 | 4.41 | 5.91 | 0.738 | 3.37 | 4.11 |
| 16 | 0.0633 | 1.38 | 1.44 | 1.60 | 4.60 | 6.20 | 0.786 | 3.54 | 4.32 |
| 17 | 0.0556 | 1.24 | 1.30 | 1.71 | 4.85 | 6.57 | 0.914 | 4.26 | 5.18 |
| 18 | 0.0676 | 1.51 | 1.57 | 1.97 | 5.61 | 7.58 | 1.08 | 5.56 | 6.64 |
| 19 | 0.0747 | 1.36 | 1.44 | 2.05 | 5.80 | 7.85 | 1.34 | 7.06 | 8.39 |
| 20 | 0.105 | 2.23 | 2.34 | 2.43 | 8.38 | 10.8 | 1.81 | 9.67 | 11.5 |
| 21 | 0.121 | 2.37 | 2.50 | 2.82 | 10.5 | 13.3 | 2.40 | 12.7 | 15.1 |
| 22 | 0.168 | 2.76 | 2.93 | 3.55 | 12.8 | 16.4 | 2.90 | 15.6 | 18.5 |
| 23 | 0.2068 | 2.89 | 3.08 | 4.62 | 15.9 | 20.6 | 3.37 | 19.9 | 23.3 |
| 24 | 0.256 | 2.85 | 3.11 | 7.05 | 28.2 | 35.2 | 3.71 | 18.5 | 22.2 |
| 25 | 0.323 | 2.74 | 3.06 | 9.29 | 39.6 | 48.9 | 6.04 | 19.8 | 25.9 |
