# Supplementary material for: Feeding Mechanics in Spinosaurid Theropods and Extant Crocodilians
Source: PLoS One. 2013 May 28;8(5):e65295. doi: 10.1371/journal.pone.0065295 (PMC3665537; doi:10.1371/journal.pone.0065295)
Supplement: Table S2 — Resistances to bending and torsion in size-corrected, crocodilian upper jaws. All values are metres ×10−07. (DOC) [file pone.0065295.s002.doc]

**Table S2. Resistances to bending and torsion in size-corrected, crocodilian rostra.** All values are metres x10^-07^

| Slice | Alligator | | | Gharial | | | *M. cataphractus* | | |
| --- | --- | --- | --- | --- | --- | --- | --- | --- | --- |
|  | Ix | Iy | J | Ix | Iy | J | Ix | Iy | J |
| 1 | 0.091 | 0.126 | 0.217 | 0.0181 | 0.180 | 0.198 | 0.0961 | 0.141 | 0.237 |
| 2 | 4.89 | 26.8 | 31.7 | 0.762 | 2.71 | 3.47 | 1.20 | 9.30 | 10.5 |
| 3 | 4.81 | 81.6 | 86.4 | 1.09 | 14.1 | 15.2 | 1.71 | 10.2 | 12.0 |
| 4 | 7.06 | 142 | 149 | 2.66 | 19.0 | 21.7 | 2.90 | 8.92 | 11.8 |
| 5 | 6.42 | 160 | 166 | 1.67 | 8.15 | 9.82 | 1.67 | 4.97 | 6.64 |
| 6 | 5.21 | 99.8 | 105 | 0.984 | 2.91 | 3.90 | 1.40 | 2.16 | 3.56 |
| 7 | 7.15 | 168 | 176 | 1.05 | 2.79 | 3.84 | 1.88 | 4.76 | 6.64 |
| 8 | 7.59 | 198 | 206 | 1.25 | 3.10 | 4.35 | 2.20 | 5.90 | 8.10 |
| 9 | 10.8 | 303 | 314 | 1.21 | 3.22 | 4.43 | 2.15 | 5.74 | 7.90 |
| 10 | 13.5 | 410 | 423 | 1.11 | 2.96 | 4.06 | 2.32 | 7.98 | 10.3 |
| 11 | 16.6 | 469 | 485 | 1.19 | 3.37 | 4.56 | 2.43 | 9.54 | 12.0 |
| 12 | 18.2 | 556 | 574 | 1.17 | 3.33 | 4.50 | 2.72 | 12.9 | 15.6 |
| 13 | 18.8 | 561 | 580 | 1.28 | 3.8 | 5.08 | 3.78 | 20.9 | 24.7 |
| 14 | 18.2 | 482 | 500 | 1.35 | 4.19 | 5.54 | 3.51 | 18.3 | 21.8 |
| 15 | 16.1 | 381 | 397 | 1.51 | 4.41 | 5.91 | 2.93 | 13.4 | 16.3 |
| 16 | 15.6 | 340 | 356 | 1.60 | 4.60 | 6.20 | 3.12 | 14.0 | 17.1 |
| 17 | 13.7 | 307 | 320 | 1.71 | 4.85 | 6.57 | 3.62 | 16.9 | 20.5 |
| 18 | 16.7 | 372 | 388 | 1.97 | 5.61 | 7.58 | 4.29 | 22.0 | 26.3 |
| 19 | 18.4 | 336 | 354 | 2.05 | 5.80 | 7.85 | 5.29 | 28.0 | 33.3 |
| 20 | 26.0 | 550 | 576 | 2.43 | 8.38 | 10.8 | 7.17 | 38.3 | 45.5 |
| 21 | 29.9 | 585 | 615 | 2.82 | 10.5 | 13.3 | 9.50 | 50.5 | 60.0 |
| 22 | 41.5 | 682 | 723 | 3.55 | 12.8 | 16.4 | 11.5 | 61.6 | 73.1 |
| 23 | 50.7 | 710 | 761 | 4.62 | 15.9 | 20.6 | 13.4 | 78.8 | 92.2 |
| 24 | 63.1 | 704 | 767 | 7.05 | 28.2 | 35.2 | 14.7 | 73.2 | 87.9 |
| 25 | 79.7 | 675 | 754 | 9.29 | 39.6 | 48.9 | 23.9 | 78.6 | 103 |
