# Supplementary material for: Feeding Mechanics in Spinosaurid Theropods and Extant Crocodilians
Source: PLoS One. 2013 May 28;8(5):e65295. doi: 10.1371/journal.pone.0065295 (PMC3665537; doi:10.1371/journal.pone.0065295)
Supplement: Table S3 — Absolute values for resistances to bending and torsion in dinosaurian and crocodilian rostra. All values are metres ×10−07. (DOC) [file pone.0065295.s003.doc]

**Table S3. Absolute values for resistances to bending and torsion in dinosaurian and crocodilian rostra.** All values are metres x10^-07^.

| Slice | *Spinosaurus* | | | *Baryonyx* | | | Alligator | | | Gharial | | | *M. cataphractus* | | |
| --- | --- | --- | --- | --- | --- | --- | --- | --- | --- | --- | --- | --- | --- | --- | --- |
|  | Ix | Iy | J | Ix | Iy | J | Ix | Iy | J | Ix | Iy | J | Ix | Iy | J |
| 1 | 0.152 | 0.168 | 0.320 | 1.54 | 1.97 | 3.51 | 0.00140 | 0.00168 | 0.00308 | 0.0280 | 0.220 | 0.248 | 0.0290 | 0.0383 | 0.0674 |
| 2 | 15.1 | 14.2 | 29.3 | 26.9 | 28.0 | 54.9 | 0.0422 | 0.244 | 0.286 | 0.690 | 2.63 | 3.32 | 0.148 | 0.592 | 0.739 |
| 3 | 16.4 | 17.9 | 34.3 | 34.0 | 34.1 | 68.0 | 0.0217 | 0.490 | 0.512 | 1.35 | 16.6 | 17.9 | 0.273 | 2.81 | 3.08 |
| 4 | 16.6 | 11.0 | 27.5 | 23.8 | 24.1 | 48.0 | 0.0367 | 0.721 | 0.757 | 2.00 | 18.3 | 20.3 | 0.570 | 3.61 | 4.18 |
| 5 | 11.5 | 6.65 | 18.2 | 16.4 | 23.9 | 40.3 | 0.0369 | 0.624 | 0.661 | 2.43 | 18.7 | 21.1 | 0.837 | 3.35 | 4.19 |
| 6 | 8.58 | 5.13 | 13.7 | 10.1 | 27.8 | 37.9 | 0.0546 | 1.16 | 1.22 | 1.72 | 7.38 | 9.10 | 0.666 | 2.06 | 2.73 |
| 7 | 10.8 | 5.75 | 16.5 | 28.0 | 74.5 | 102 | 0.0638 | 1.56 | 1.63 | 1.12 | 3.17 | 4.29 | 0.364 | 0.708 | 1.07 |
| 8 | 12.7 | 6.73 | 19.4 | 54.2 | 133 | 187 | 0.0867 | 2.19 | 2.28 | 1.20 | 3.24 | 4.44 | 0.344 | 0.570 | 0.914 |
| 9 | 0.152 | 0.168 | 0.320 | 1.54 | 1.97 | 3.51 | 0.00140 | 0.00168 | 0.00308 | 0.0280 | 0.220 | 0.248 | 0.0290 | 0.0383 | 0.0674 |
| 10 | 15.1 | 14.2 | 29.3 | 26.9 | 28.0 | 54.9 | 0.0422 | 0.244 | 0.286 | 0.690 | 2.63 | 3.32 | 0.148 | 0.592 | 0.739 |
| 11 | 16.4 | 17.9 | 34.3 | 34.0 | 34.1 | 68.0 | 0.0217 | 0.490 | 0.512 | 1.35 | 16.6 | 17.9 | 0.273 | 2.81 | 3.08 |
| 12 | 16.6 | 11.0 | 27.5 | 23.8 | 24.1 | 48.0 | 0.0367 | 0.721 | 0.757 | 2.00 | 18.3 | 20.3 | 0.570 | 3.61 | 4.18 |
| 13 | 11.5 | 6.65 | 18.2 | 16.4 | 23.9 | 40.3 | 0.0369 | 0.624 | 0.661 | 2.43 | 18.7 | 21.1 | 0.837 | 3.35 | 4.19 |
| 14 | 8.58 | 5.13 | 13.7 | 10.1 | 27.8 | 37.9 | 0.0546 | 1.16 | 1.22 | 1.72 | 7.38 | 9.10 | 0.666 | 2.06 | 2.73 |
| 15 | 10.8 | 5.75 | 16.5 | 28.0 | 74.5 | 102 | 0.0638 | 1.56 | 1.63 | 1.12 | 3.17 | 4.29 | 0.364 | 0.708 | 1.07 |
