# Supplementary material for: Feeding Mechanics in Spinosaurid Theropods and Extant Crocodilians
Source: PLoS One. 2013 May 28;8(5):e65295. doi: 10.1371/journal.pone.0065295 (PMC3665537; doi:10.1371/journal.pone.0065295)
Supplement: Table S4 — Resistances to bending and torsion in size-corrected dinosaurian and crocodilian rostra. All values are metres ×10−07. (DOC) [file pone.0065295.s004.doc]

**Table S4. Resistances to bending and torsion in size-corrected dinosaurian and crocodilian rostra. All values are metres x10^-07^.**

| Slice | *Spinosaurus* | | | *Baryonyx* | | | Alligator | | | Gharial | | | *M. cataphractus* | | |
| --- | --- | --- | --- | --- | --- | --- | --- | --- | --- | --- | --- | --- | --- | --- | --- |
|  | Ix | Iy | J | Ix | Iy | J | Ix | Iy | J | Ix | Iy | J | Ix | Iy | J |
| 1 | 0.0433 | 0.0479 | 0.0912 | 1.93 | 2.48 | 4.42 | 0.344 | 0.415 | 0.759 | 0.028 | 0.220 | 0.248 | 0.115 | 0.152 | 0.267 |
| 2 | 4.30 | 4.03 | 8.33 | 33.9 | 35.3 | 69.2 | 10.4 | 60.1 | 70.5 | 0.690 | 2.63 | 3.32 | 0.586 | 2.34 | 2.93 |
| 3 | 4.67 | 5.11 | 9.78 | 42.8 | 42.9 | 85.6 | 5.36 | 121 | 126 | 1.35 | 16.6 | 17.9 | 1.08 | 11.1 | 12.2 |
| 4 | 4.73 | 3.12 | 7.85 | 30.0 | 30.4 | 60.4 | 9.05 | 178 | 187 | 2.00 | 18.3 | 20.3 | 2.26 | 14.3 | 16.6 |
| 5 | 3.28 | 1.89 | 5.18 | 20.7 | 30.1 | 50.7 | 9.11 | 154 | 163 | 2.43 | 18.7 | 21.1 | 3.32 | 13.3 | 16.6 |
| 6 | 2.44 | 1.46 | 3.90 | 12.7 | 35.0 | 47.7 | 13.5 | 287 | 300 | 1.72 | 7.38 | 9.10 | 2.64 | 8.16 | 10.8 |
| 7 | 3.07 | 1.64 | 4.71 | 35.3 | 93.8 | 129 | 15.7 | 385 | 401 | 1.12 | 3.17 | 4.29 | 1.44 | 2.81 | 4.25 |
| 8 | 3.61 | 1.92 | 5.53 | 68.2 | 167 | 236 | 21.4 | 541 | 562 | 1.20 | 3.24 | 4.44 | 1.36 | 2.26 | 3.62 |
| 9 | 0.0433 | 0.0479 | 0.0912 | 1.93 | 2.48 | 4.42 | 0.344 | 0.415 | 0.759 | 0.028 | 0.22 | 0.248 | 0.115 | 0.152 | 0.267 |
| 10 | 4.30 | 4.03 | 8.33 | 33.9 | 35.3 | 69.2 | 10.4 | 60.1 | 70.5 | 0.690 | 2.63 | 3.32 | 0.586 | 2.34 | 2.93 |
| 11 | 4.67 | 5.11 | 9.78 | 42.8 | 42.9 | 85.6 | 5.36 | 121 | 126 | 1.35 | 16.6 | 17.9 | 1.08 | 11.1 | 12.2 |
| 12 | 4.73 | 3.12 | 7.85 | 30.0 | 30.4 | 60.4 | 9.05 | 178 | 187 | 2.00 | 18.3 | 20.3 | 2.26 | 14.3 | 16.6 |
| 13 | 3.28 | 1.89 | 5.18 | 20.7 | 30.1 | 50.7 | 9.11 | 154 | 163 | 2.43 | 18.7 | 21.1 | 3.32 | 13.3 | 16.6 |
| 14 | 2.44 | 1.46 | 3.9 | 12.7 | 35.0 | 47.7 | 13.5 | 287 | 300 | 1.72 | 7.38 | 9.10 | 2.64 | 8.16 | 10.8 |
| 15 | 3.07 | 1.64 | 4.71 | 35.3 | 93.8 | 129 | 15.7 | 385 | 401 | 1.12 | 3.17 | 4.29 | 1.44 | 2.81 | 4.25 |
